# Incident SARS-CoV-2 Infection and a Shared Latrine

**DOI:** 10.4269/ajtmh.20-0793

**Published:** 2020-07-22

**Authors:** Oscar H. Del Brutto, Aldo F. Costa, Héctor H. García

**Affiliations:** 1 School of Medicine, Universidad Espíritu Santo, Ecuador, Samborondón, Ecuador;; 2 Community Center, The Atahualpa Project, Atahualpa, Ecuador;; 3 Department of Microbiology, Center for Global Health, Universidad Peruana Cayetano Heredia, Lima, Perú

In Atahualpa, a rural Ecuadorian village, a cohort study has evaluated most adult villagers for factors associated with chronic diseases along the past 9 years. The SARS-Cov-2 pandemic struck Atahualpa in March 2020, as shown by an almost 3-fold excess mortality during April and May, and by a significant number of symptomatic cases.

**Figure 1.**
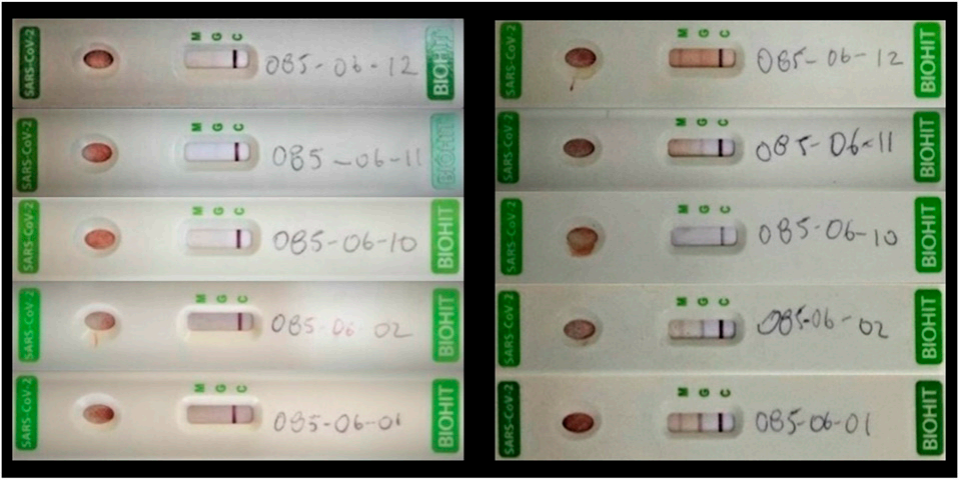
Serological results of the five household members of house B showing negative results to SARS-CoV-2 antibodies during the first survey (left panel). At the time of the second survey (1 month later), four of them seroconverted.

**Figure 2.**
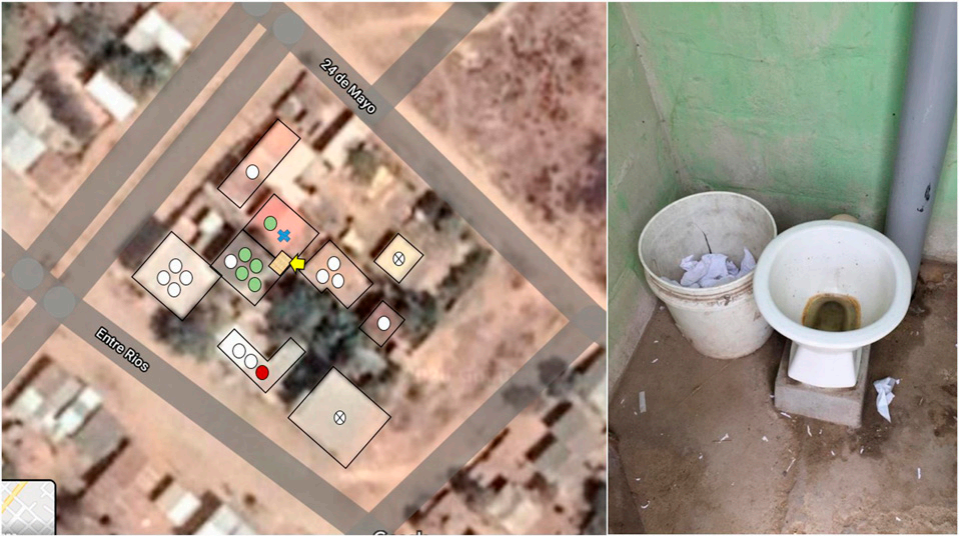
Left panel: Satellite view (Google Earth, Google Inc., Mountain View, CA) of the block where the described cases occurred. Yellow arrow points to the shared latrine. The blue cross refers to the visitor (index case) and the green dot in the same house to his seroconverted grandmother. Behind house A, house B shows four seroconverted family members (green dots) and the one that remained seronegative (white dot). The other white dots across other houses of the block refer to individuals who were seronegative at the first and the second surveys, and the red dot to a single individual who was seropositive at the first survey. Right panel: Shared latrine under poor hygienic conditions.

In a recent serosurvey, we found that the use of open latrines (instead of flushing toilet systems) was significantly associated with seropositivity to SARS-CoV-2 on lateral flow-based antibody testing (BIOHIT Health Care Ltd., Cheshire, United Kingdom), suggesting a contributory role for fecal–oral transmission of the disease, as previously proposed by others.^1^ The baseline study was followed by a second round of testing 4 weeks later to assess the incidence of infection. Tests were stored and managed as per manufacturer instructions, and tests were performed in the field by a trained medical doctor. Here, we present a cluster of incident cases of SARS-CoV-2 involving a woman who lived alone (house A), and a five-member family (house B) who were seronegative during the first survey. These families were not related to each other but shared a latrine located between both houses. Two weeks after our baseline serosurvey, a 22-year-old grandson of the old woman moved into Atahualpa from Guayaquil (a heavily infected urban center), staying at her house and using the shared latrine. None of the families refers any other direct interaction or social gathering. During the second serosurvey, the woman in house A seroconverted to positive, and her visiting grandson also resulted seropositive. Likewise, four of the five family members of house B became seropositive (Figure 1). There were no other incident cases in the entire block, where only one person in a distant house had tested positive at baseline, and several other inhabitants of other houses remained seronegative (Figure 2, left). We hypothesize that the visitor was likely infected when he arrived, infected his grandmother by living together, and then the infection was spread to the neighbor’s house by fecal contamination of one of the members while using the latrine, which was poorly maintained (Figure 2, right). Certainly, we cannot rule out other interpersonal contacts, but—as previously mentioned—neither of the families refer interaction. Similarly, we cannot conclude on whether the individual who did not seroconvert was less exposed or had any sort of reduced individual predisposition to infection. This cluster of new infections provides circumstantial evidence that latrines (ergo, fecal contamination) may act as a source of infection. Poor hygienic conditions are frequent in rural regions of developing countries and may contribute to the spread of SARS-CoV-2.^2^
